# Role of New Potential Biomarkers in the Risk of Thromboembolism in Atrial Fibrillation

**DOI:** 10.3390/jcm11040915

**Published:** 2022-02-09

**Authors:** Mario Piergiulio Pezzo, Antonella Tufano, Massimo Franchini

**Affiliations:** 1 Department of Transfusion Medicine and Hematology, Carlo Poma Hospital, 46100 Mantova, Italy; mariopiergiulio.pezzo@asst-mantova.it; 2 Department of Clinical Medicine and Surgery, University of Naples “Federico II”, 80131 Naples, Italy; atufano@unina.it

**Keywords:** atrial fibrillation, biomarkers, stroke

## Abstract

Ischemic stroke risk in atrial fibrillation differs from patient to patient, depending on numerous variables. Many attempts have been made to translate this difference into simple numbers and to compare it to the hemorrhagic risk of anticoagulation. Different clinical scores have been studied to define a clear strategy. One score, the CHA_2_DS_2_-VASc score, has been extensively and successfully applied worldwide. Nevertheless, it is not yet the “perfect instrument”. Many proposals have been made to integrate its clinical parameters with some biomarkers to improve its predictive power. This short review describes some of these biomarkers and their possible implications in potentiating the efficacy of clinical scores.

## 1. Introduction

Anticoagulant therapy is always a balance between hemorrhagic risk and therapy benefits. This is especially true for atrial fibrillation, considering that many variables play a role in creating different profiles of risk for ischemic stroke.

Worldwide, the cardiac arrhythmia with the highest prevalence is atrial fibrillation (AF), a condition that results in a significantly increased risk of ischemic stroke (IS). Instruments to stratify the risk of IS are widely used to guide decisions regarding anticoagulation treatment in patients with non-valvular AF (NVAF) [1,2]. The CHA_2_DS_2_-VASc score, which is widely validated, is currently the available “state of the art” instrument. Nevertheless, its properties and decisional standpoints are rather “granular”, and there is considerable overlap between the risk predicted by different score values. A possible refinement could be to find one or more biomarkers to “integrate” the score and make it applicable with higher risk discrimination.

## 2. CHA_2_DS_2_-VASc Score

The acronym stands for Congestive heart failure (one point if present), High blood pressure (one point), Age (two points if above 75 years), Diabetes (one point), previous Stroke (two points), Vascular disease (one point), Age (one point if between 65 and 74 years), Sex (one point if female). According to Lip and colleagues [1] and Olesen and colleagues [2], the resulting score is associated with different IS risk profiles (Table 1).

Although the CHA_2_DS_2_-VASc score is a simple and practical instrument, it does have some limitations. For example, the contributions of its individual components to IS risk in patients with NVAF are unequal. Still, most components are assigned an equal value, and only two risk factors, age and prior stroke (or transient ischemic attack) are given a different weight [3,4].

Moreover, there may be racial or ethnic differences in the prediction of IS in NVAF, and some other known risk factors or biomarkers are not incorporated in the score, such that the predictive performance is suboptimal in selected populations (e.g., patients with renal failure) [5,6,7]. Finally, a recent systematic review showed that the predictive power of the CHA_2_DS_2_-VASc score is not perfect (c-statistic of 0.6–0.7) [8]. 

## 3. ABC Stroke Risk Score

The ABC stroke risk [9] score deserves a particular mention. Although newer and less known than the CHA_2_DS_2_-VASc score, some studies have demonstrated it to overcome the CHA_2_DS_2_-VASc predictive results [10]. This score underlines once again the importance of biomarkers in assessing the IS risk of Atrial Fibrillation in different categories of patients.

The acronym stands for Age, Biomarkers, and Clinical history. It’s a normogram-based score to predict the stroke risk in AF patients.

The considered biomarkers are Cardiac Troponins and N-Terminal pro-B-type natriuretic peptide (NT-proBNP).

## 4. Biomarkers

An ideal biomarker should be simple and practical, have a high sensitivity and be inexpensive. 

### 4.1. Clinical Biomarkers 

Many clinical biomarkers have been proposed in the literature to be added to the score to increase its sensitivity and specificity. Based on current evidence, some clinical markers (e.g., non-paroxysmal type of AF, carotid plaque) and some circulating biomarkers (e.g., cardiac troponin, N-terminal pro-B-type natriuretic protein [BT-proBNP], and D-dimer) are promising for use in IS prediction in patients with NVAF because it is both practical and simple to determine them.

A recent, comprehensive review reports many of the potential biomarkers are divided into “clinical” and “circulating” [11]. Below is a concise list of potential clinical biomarkers, although we focus only on circulating biomarkers. The evidence of their predictive value is strong for some of them, whereas for others, the evidence is more controversial [12].

- AF characteristics: type, duration, and burden
- Cardiac imaging:
  o Left atrial appendage (LAA) morphology, the position of the LAA orifice, LAA flow velocity
  o Left atrium diameter, atrial fibrosis, left atrial strain
  o Left ventricle relative wall thickness, left ventricular ejection fraction
  o Epicardial fat thickness, left atrium spontaneous echo contrast
- ECG:
  o P wave terminal force in lead V1
  o P wave duration
  o Maximum P wave
  o Advanced interatrial block
- Atherosclerosis:
  o Carotid intima-media thickness
  o Carotid plaques
  o Flow-mediated dilation

### 4.2. Circulating Biomarkers 

#### 4.2.1. Cardiac Troponins

Troponin is a marker of cardiac necrosis that has been extensively used for decades. It is the most widely used biomarker in cardiovascular disease. Its value in detecting myocardial damage is undisputed, and it can be used to make many important pathophysiological considerations regarding the disease evolution and prognosis

Evidence shows that troponin could be used to predict IS in patients with AF. For example, post-hoc analyses of the clinical trials ARISTOTLE [13], RE-LY [14], and ENGAGE AF-TIMI [15] indicated a positive association between cardiac troponin 1/troponin T levels and the risk of thromboembolism or IS. 

V. Roldan et al. demonstrated in a large ‘real-world’ cohort of anticoagulated AF patients that the levels of both high-sensitivity troponin T and high-sensitivity interleukin-6 provided prognostic information that was complementary to that of clinical risk scores for predicting long-term cardiovascular events and death. Therefore, these biomarkers could help to refine risk stratification in AF [16].

A meta-analysis published in 2020 suggested that high-sensitivity cardiac troponin can be considered a biomarker for the risk of incident stroke, with its effect size differing in different subgroups [17].

#### 4.2.2. BNP and NT-proBNP

B-type natriuretic peptide (BNP) and NT-proBNP are markers of heart failure, secreted in response to increased end-diastolic pressure and/or volume expansion. NT-proBNP results from the enzymatic cleavage of the BNP produced by cardiac myocytes.

Due to atrial dysfunction, their increment may be predictive of higher IS risk [12].

Analyses of the results of the ARISTOTLE [13], RE-LY [14], and ENGAGE AF-TIMI 48 [15] trials demonstrated that NT-proBNP levels were independently associated with an increased risk of IS, and, thus, adding NT-proBNP to the CHA_2_DS_2_-VASc score could improve this latter’s predictive power.

In a real-world group of patients with AF receiving anticoagulant treatment, NT-proBNP levels provided complementary prognostic information to the CHA_2_DS_2_-VASc score for predicting stroke/systemic embolism. NT-proBNP was also predictive of all-cause mortality. Adding NT-proBNP to the CHA_2_DS_2_-VASc score increased the ability of the score to predict the risk of IS/systemic embolism in anticoagulated patients with AF by 17% [18].

#### 4.2.3. D-Dimer

D-dimer is a degradation product of cross-linked fibrin and a biomarker of activated coagulation and fibrinolysis. Higher levels of D-dimer have been associated with an increased probability of IS [6,19,20,21,22].

A study of 323 patients with NVAF who were not receiving anticoagulation showed that D-dimer level is positively correlated with IS risk stratification but does not have a predictive value for the occurrence of IS in patients with NVAF [23]. Thus, dynamic detection of D-dimer levels might be necessary for patients with NVAF. 

#### 4.2.4. Red Cell Distribution Width 

An increased red cell distribution width (RDW) value, due to impaired erythropoiesis, may be associated with thromboembolic events in patients with NVAF and was indeed found to be independently associated with such events in patients with NVAF [24].

#### 4.2.5. Neutrophil-to-Lymphocyte Ratio

The neutrophil-to-lymphocyte ratio (NLR) is an inexpensive, readily available marker of inflammation. It was associated with an increased risk of long-term mortality in patients admitted to hospital with acute decompensated heart failure, in whom it could be used to aid risk stratification [25]. NLR was also found to be directly associated with stroke risk in a large cohort of AF patients. Adding NLR to the CHA_2_DS_2_-VASc score increased the area under the curve from 0.627 (95% confidence interval 0.612–0.643) to 0.635 (0.619–0.651) (*p* = 0.037) [26]. 

#### 4.2.6. Mean Platelet Volume

Mean platelet volume (MPV) indicates the intensity of an inflammatory process and correlates with the risk of thrombotic complications. A study of NVAF patients provided evidence that MPV is an independent predictor of IS in such a population. The combination of the CHA_2_DS_2_-VASc score and MPV had a sensitivity of 72.1% for predicting IS and a specificity of 81.5% [27]. 

#### 4.2.7. Von Willebrand Factor

Von Willebrand factor (VWF) levels were found to be increased in patients with AF in early studies [28], and in subsequent studies [29,30], they were found to be a predictor of IS and an independent risk factor.

#### 4.2.8. Plasma Fibrinogen

As noted for VWF, plasma fibrinogen levels were found high in AF patients [31] and positively associated with leukoaraiosis and periventricular hyperintensity in patients with stroke and AF [32].

Prothrombin fragment 1.2 is an index of thrombin generation. It has been demonstrated that thrombin generation increases during AF despite the use of anticoagulants [33]. Though F 1.2 levels and thrombin generation are clearly implicated in the thromboembolic process, their role in predicting or improving the IS risk stratification in AF needs to be investigated more deeply.

#### 4.2.9. Cholesterol and Lipoproteins

Low-density lipoprotein-cholesterol (LDL-C) was identified as an independent predictor of IS in patients with NVAF in one study [34], but others have not confirmed this result. However, total cholesterol and LDL-C were higher in patients with NVAF who suffered IS than in patients who did not [4].

The ratio of LDL-C to high-density lipoprotein-cholesterol was found to predict IS in a case control study [35] while higher levels of lipoprotein(a) (≥30 mg/dL) seemed to be associated with thromboembolic events [36] although other studies contradict these data.

### 4.3. Biomarkers of Inflammation

#### 4.3.1. C-Reactive Protein

There is considerable evidence that AF is associated with an inflammatory state. 

Dernellis et al. [37] described that the occurrence of paroxysmal AF in patients with a high C-reactive protein level is associated with enlargement of the left atrium and depression of left atrial contractile function independently of left ventricular hypertrophy and function.

Lip et al. [38] found that, among AF patients, C-reactive protein level was positively correlated with stroke risk and related to risk factors for stroke and prognosis (mortality, vascular events).

#### 4.3.2. Uricemia

Hyperuricemia can increase the risk of AF [39]. Numa et al. [40] described that the serum uric acid level was associated with thromboembolic risk on transesophageal echocardiography in patients with NVAF at low-intermediate risk according to stratification by clinical risk factors.

#### 4.3.3. Soluble CD40L

Soluble CD40L is an 18-KDa trimer that is shed by activated T lymphocytes and platelets and is considered a marker of inflammation and thrombosis in several diseases. Increased levels have been associated with the presence of left atrial thrombus [41] and cerebrovascular events [42].

#### 4.3.4. Homocysteine

In a series of studies, hyperhomocysteinemia has been found to be independently associated with left atrial thrombus formation in IS patients with NVAF and with a history of IS in NVAF patients hospitalized for cardiac reasons [43,44].

#### 4.3.5. GDF-15, TMAO, IL-1ra, IL-6

Growth differentiation factor-15 (GDF-15) is a transforming growth factor beta superfamily member. Physiologically, GDF-15, a peptide hormone, is expressed at low concentrations in numerous tissues. GDF-15 is overexpressed during and after many pathological conditions, including tissue injury and inflammation, in order to exert a protective function. Elevated GDF-15 levels may indicate a significantly increased risk of left atrial/LAA thrombus in NVAF patients. They could, therefore, potentially be a useful adjunct in discriminating left atrial/LAA thrombus in such patients [45].

Trimethylamine N-oxide (TMAO) is a small amine oxide that is generated by the microbial metabolism of choline, betaine, and carnitine in the gut. There is a report of a positive correlation between elevated plasma levels of TMAO and an increased risk of major adverse cardiovascular events and death. In a recent work [46], univariate and multivariate logistic regression analyses indicated that TMAO was an independent predictor in IS. The level of TMAO was correlated with the CHA_2_DS_2_-VASc score. Since TMAO is thought to be an independent predictor of IS, it could potentially refine stroke stratification in patients with AF.

The causal role of interleukins in the pathogenesis of vascular diseases has not been fully elucidated. However, a recent article [47] described an inverse relationship of interleukin-6 and a direct one of interleukin-1 receptor antagonist with cardio-embolic stroke.

### 4.4. Other Biomarkers

#### 4.4.1. Galectin-3

Galectin-3, a lectin that binds β-galactosides, is involved in cardiac remodeling and fibrosis through its effect of activating macrophages and fibroblasts. Various studies have documented that increased levels of circulating galectin-3 are associated with AF-related IS and LAA thrombus formation [48,49]. 

#### 4.4.2. ST-2

Suppression of tumorigenicity 2 (ST2) is a member of the interleukin-1 receptor family and circulating soluble ST2 concentrations have been suggested to be a biomarker of cardiovascular stress and fibrosis and were independently and significantly related to AF recurrence in patients with NVAF [50].

#### 4.4.3. Protein Biomarker Discovery Platforms

Protein concentrations in blood are more closely linked to disease phenotype than any other biomarker in blood. New protein biomarker discovery platforms as Olink Explore or SomaLogic that can sustain high throughput, though maintaining the performance of the “state of the art” manual methods, can be successfully used to investigate the continuously evolving field of circulating biomarkers and their practical applications [11]. 

## 5. Conclusions

An increasing number of new biomarkers that may predict AF-related IS risk and have prognostic value are currently under investigation. Some of them are strong indicators of progressive cardiac structural remodeling, while others only demonstrate the “burden” of the disease. 

Biomarkers of hemodynamic stress, myocardial injury, and coagulation activity are eligible indicators of thromboembolic risk. Nevertheless, it is still unclear how the information that they provide can be exploited, and their predictive value is often applied only to sub-groups. Some of them reflect the pathophysiological processes of AF, whereas others are only markers of severity or comorbidity related to an increased risk of AF, recurrence, or mortality. At present, their additional predictive value is small, and their clinical usefulness and net benefit over current clinical scores have not been clearly established.

The European guidelines for AF management suggest that biomarkers can be useful for predicting stroke, but this conclusion is based on very selected clinical trials and probably cannot be simply extended to a “real-world” situation. Biomarkers do not currently appear to add better prognostic discrimination to lower values of the CHA_2_DS_2_-VASc score. In brief, more studies are needed to determine unequivocally whether adding one or more biomarkers would potentiate the predictive power of current clinical scores and whether the advantage could be extended to the “real world”.

## Figures and Tables

**Table 1:** CHA_2_DS_2_-VASc score relative risk.

Score	IS % Risk (per Year)
0	0.78 (0.58–1.04)
1	2.01 (1.70–2.36)
2	3.71 (3.36–4.09)
3	5.92 (5.53–6.34)
4	9.27 (8.71–9.86)
5	15.26 (14.35–16.24)
6	19.74 (18.21–21.41)
7	21.50 (18.75–24.64)
8	22.38 (16.29–30.76)
9	23.64 (10.62–52.61)
